# Antibiotic adjuvants: identification and clinical use

**DOI:** 10.1111/1751-7915.12044

**Published:** 2013-02-28

**Authors:** Patricia Bernal, Carlos Molina-Santiago, Abdelali Daddaoua, María A Llamas

**Affiliations:** Estación Experimental del Zaidín, Consejo Superior de Investigaciones Científicas (CSIC)18008, Granada, Spain

The discovery of penicillin by Alexander Fleming in 1928 changed the course of medicine. Since then, antibiotics have represented virtually the only effective treatment option for bacterial infections. However, their efficacy has been seriously compromised by over-use and misuse of these drugs, which have led to the emergence of bacteria that are resistant to many commonly used antibiotics. Bacteria present three general categories of antibiotic resistance: acquired, intrinsic and adaptive (Alekshun and Levy, 2007). Acquired resistance is the result of mutations in chromosomal genes or the incorporation of new genetic material (plasmids, transposons, integrons, naked DNA) by horizontal gene transfer. It provides selective advantage in the presence of antimicrobial compounds and it is passed on to progeny resulting in the emergence of antibiotic-resistant strains. Bacteria have an extraordinary ability to acquire antibiotic resistance, which is best understood from an evolutionary perspective. Thus, while the use of antibiotics as therapeutics started less than 70 years ago, bacterial resistance mechanisms have co-evolved with natural antimicrobial compounds for billions of years (D'Costa *et al*., 2011). Bacterial intrinsic resistance to antibiotics is, in contrast to acquired resistance, not related to antibiotic selection but to the specific characteristics of the bacteria. Gram-negative bacteria are for example resistant to many antibiotics due to the presence of a lipopolysaccharide-containing outer membrane with low permeability that functions as an extra barrier preventing the entrance of antibiotics into the cell. Furthermore, many bacteria contain efflux pumps that pump antibiotics out of the cell and thereby decrease their effectiveness. Finally, adaptive resistance involves a temporary increase in the ability of a bacterium to survive an antibiotic, mainly as the result of alterations in gene and/or protein expression triggered by environmental conditions (i.e. stress, nutrient conditions, growth state, subinhibitory levels of the antibiotic) (Poole, 2012). In contrast to intrinsic and acquired resistance mechanisms, which are stable and can be transmitted on to the progeny, adaptive resistance is transient and usually reverts upon the removal of the inducing condition.

The combination of these antibiotic resistance mechanisms has led to the emergence of multidrug-resistant pathogens, which are a serious threat for medical care. Among other strategies, the discovery or development of new antibiotic agents had been thought to be a solution to overcome the deficiencies of the existing ones. However, development and marketing approval of new antibiotics have not kept pace with the increasing public health threat of bacterial drug resistance. An alternative to the development of new antibiotics is to find potentiators of the already existing ones, a less expensive alternative to the problem (Ejim *et al*., 2011; Kalan and Wright, 2011). Potentiators of antibiotic activity are known as antibiotic adjuvants. These compounds are active molecules, preferably with non-antibiotic activity, that in combination with antibiotics enhance the antimicrobial activity of the latter. Combinations of two antibiotics are also considered adjuvants when their effect is synergistic (i.e. the co-administration of the two drugs has a significantly greater effect than that of each antibiotic alone). Antibiotic adjuvants can function either by reversing resistance mechanisms in naturally sensitive pathogens or by sensitizing intrinsic resistant strains. Identification of new molecules that can function as adjuvants is currently an important topic of research. In this context, Taylor and colleagues have recently published a work aimed to identify molecules that potentiate the antimicrobial activity of antibiotics commonly used against Gram-positive bacteria but that have, however, little or no effect on Gram-negative pathogens (Taylor *et al*., 2012). Using the Gram-negative bacterium *Escherichia coli* as model in combination with the aminocoumarin antibiotic novobiocin, the authors set up and performed a forward chemical genetic screen with a library of 30 000 small molecules. Three rounds of selection in which molecules that did not enhance novobiocin activity, that had intrinsic antibacterial activity, or that had undesirable secondary effects were discarded, identified four new compounds that increase the antimicrobial activity of novobiocin and other ‘Gram-positive’ antibiotics against *E. coli*. All identified molecules alter bacterial cell shape by blocking cytoskeleton proteins (i.e. MerB) and/or peptidoglycan biosynthesis, and act synergistically with the antibiotic. Authors conclude that cell shape alterations likely disturb the influx/efflux machinery of Gram-negative bacteria and thereby enable the accumulation of otherwise excluded antibiotics. This finding provides an attractive strategy to combat the intrinsic antibiotic resistance of Gram-negative bacteria and can aid the development of new therapies that enhance the activity of existing antibiotics against them.

The four compounds identified in this work potentiate antibiotic activity by affecting a vital physiological bacterial function, but potentiation of antibiotic activity can also occur by: (i) inhibition of antibiotic resistance elements; (ii) enhancement of the uptake of the antibiotic through the bacterial membrane; (iii) direct blocking of efflux pumps; and (iv) changing the physiology of resistant cells (i.e. dispersal of biofilms to planktonic cells which are more susceptible to antibiotics) (Kalan and Wright, 2011). Examples of currently used/identified antibiotic adjuvants are given in Table 1. The most successful and clinically used strategy to date has been the combination of a β-lactam antibiotic with a β-lactamase inhibitor adjuvant. The β-lactamase inhibitor enhances the action of the antibiotic by inhibiting the function of the β-lactam degrading enzyme β-lactamases. Thus, the adjuvant restores the activity of the β-lactam antibiotic against β-lactamase-producing pathogens. Three β-lactamase inhibitors have already been registered: clavulanic acid, tazobactam and sulbactam (Drawz and Bonomo, 2010) (Table 1). Clavulanic acid is mainly given in combination with the antibiotic amoxicillin, which has been commercialized as Augmentin® (Brown *et al*., 1976). Although this antimicrobial drug combination is on the market since 1981 and has been extensively used, the emergence of resistance to Augmentin in clinical isolates has been very low (Leflon-Guibout *et al*., 2000), which is another important advantage of pairing antibiotics with adjuvants. The strategy of pairing an inhibitor of antibiotic degrading enzymes with the antibiotic has also been applied against dehydropeptidase, an enzyme that degrades the β-lactam antibiotic imipenem. The adjuvant cilastatin inhibits the action of this enzyme and protects imipenem from degradation prolonging its antibacterial effect when given in combination (Balfour *et al*., 1996) (Table 1). Inhibitors for aminoglycoside-modifying enzymes and erythromycin ribosomal methylases have also been identified (Feder *et al*., 2008; Vong *et al*., 2012), but none of them has been considered sufficiently potent for further development as antibiotic adjuvants. Another way of preventing antibiotic degradation is by targeting the bacterial regulatory systems involved in the expression of antibiotic resistance genes. Bacteria respond to specific environmental signals, such as presence of antibiotics, using signal transduction mechanisms (i.e. two-component systems). Inhibition of such regulatory systems is a promising strategy for the development of antibiotic adjuvants (Lee *et al*., 2009; Nguyen *et al*., 2010). Desirable candidates for antibiotic adjuvants are also those molecules that enhance antibiotic entrance into cells. Polymyxin E, also known as colistin, is a cationic polypeptide antibiotic that interferes with the LPS and permeabilizes the outer membrane of Gram-negative bacteria. Clinical use for this antibiotic has been limited due to toxicity concerns, but at lower concentrations it has been used as adjuvant and enhances the activity of the antibiotics rifampin and vancomycin against Gram-negative pathogens (Aoki *et al*., 2009; Gordon *et al*., 2010). Molecules that prevent antibiotics from being pump out the bacterial cells are also desirable adjuvants. Generally, there are several possibilities to achieve inhibition of bacterial efflux pumps (for review see Pagès and Amaral, 2009). One of the most promising starting points is the use of substrate analogues that compete with the antibiotic for the pump since such analogues can be rationally designed (Van Bambeke *et al*., 2010) (Table 1). To date, a large number of efflux pump inhibitors have been discovered and patented (Van Bambeke *et al*., 2010; Bhardwaj and Mohanty, 2012). Although the process of commercialization of these molecules is rather slow, efflux pump inhibitors represents a promising strategy for antibiotic combination therapy. Furthermore, adjuvants can enhance antibiotic potency by changing the physiology of resistant cells. An example is by disrupting the bacterial biofilm lifestyle, in which bacteria are more resistant to antibiotic (Stewart and Costerton, 2013). Mixtures of d-amino acids have been shown to disperse biofilm of Gram-positive and Gram-negative bacteria (Kolodkin-Gal *et al*., 2010). Moreover, the combination of antibiotic with antibiofilm exopolysaccharides is also a promising strategy to enhance the antimicrobial activity of common antibiotics, having the advantage that exopolysaccharides are not toxic for human tissues (Bernal and Llamas, 2012; Rendueles *et al*., 2013).

**Table 1:** Antibiotic adjuvants

Mode of action		Adjuvant	Antibiotic	Commercial combination	Reference
Inhibition of vital physiology pathways	Complete inhibition of the folic acid biosynthetic pathway	–	Sulfamethoxazole/Trimethoprim	Septra® and Bactrim®	Reeves (1971)
	Inhibition of the synthesis and repair of the bacterial cell wall	Fosfomycin	Gentamicin, Amikacin, Ceftazidime, Cefepime, Ciprofloxacin, Levofloxacin, and Aztreonam	–	Kastoris *et al*. (2010)
	Oxidative stress outbreak	Tellurite	Ampicillin, Cefotaxime, Tetracycline, Chloramphenicol, Gentamicin	–	Molina-Quiroz *et al*. (2012)
	Cell shape alterations	Compound 1, A22, pivmecillinam, and echinomycin	Novobiocin	–	Taylor *et al*. (2012)
					
Inhibition of antibiotic resistance elements	β-lactamase inhibitors	Clavulanic acid	Amoxicillin	Augmentin®	Brown *et al*. (1976); Drawz and Bonomo (2010)
			Ticarcillin	Timentin®	Drawz and Bonomo (2010)
			Meropenem	–	Hugonnet *et al*. (2009)
		Sulbactam	Ampicillin	Unasyn®	Drawz and Bonomo (2010)
		Tazobactam	Piperacillin	Tazocin® and Zosyn®	Drawz and Bonomo (2010)
	Dehydropeptidase I inhibitor	Cilastatin	Imipenem	Tienam®	Balfour *et al*. (1996)
					
Enhance the uptake of the antibiotic through the bacterial membrane	Antibiotics that damage the cell wall improving uptake of other antibiotics	β-lactams, bacitracin, vancomycin, cycloserine	Aminoglycosides (Streptomycin, Gentamicin)	–	Chanbusarakum and Murray (1978); Moellering *et al*. (1986); Barnes *et al*. (2005)
	Binds to and interferes with the integrity of the LPS-containing outer membrane layer	Colistin (polymyxin E)	Rifampin or Vancomycin	–	Aoki *et al*. (2009); Gordon *et al*. (2010)
	Damages the bacterial membrane	Eugenol (from *Eugenia aromatic*)	Vancomycin	–	Hemaiswarya and Doble (2009)
		Phenylpropanoids	Amikacin, Ampicillin, Ciprofloxacin, Erythromycin and Vancomycin	–	Hemaiswarya and Doble (2010)
	Permeabilizes the bacterial membrane	Loperamid	Tetracyclines	–	Ejim *et al*. (2011)
					
Blocking of efflux pumps	Competitive inhibition	Tretacycline analogues	Tetracyclines	–	Van Bambeke *et al*. (2010)
		Fluoroquinolone analogues	Fluoroquinolones, Macrolides	–	Van Bambeke *et al*. (2010)
		Aminoglycoside analogues	Gentamicin	–	Van Bambeke *et al*. (2010)
					
Change the physiology of resistant cells	Dispersal of biofilms to planktonic cells	Antibiofilm exopolysaccharides	Combined with high-spectrum antibiotics	–	Rendueles *et al*. (2013)
		d-aminoacids	Combined with high-spectrum antibiotics (Ciprofloxacin, Tobramycin)	–	Kolodkin-Gal *et al*. (2010)
		Nitric Oxide (NO)	Tobramycin	–	Barraud *et al*. (2006)

In conclusion, the use of antibiotic adjuvants has two beneficial outcomes: enhancement of the antimicrobial effect and reduction of the occurrence of mutations that result in resistance. In this context, efforts to find such molecules should be intensified. Since environmental organisms are the source of most resistance genes and antibiotics (D'Costa *et al*., 2006), screens of bacterial natural products are likely to be productive in finding molecules that inhibit antibiotic resistance elements, as proven by the discovery of clavulanic acid (Brown *et al*., 1976). Additionally, a screen of a library of plant-derived compounds has also identified potentiators of antibiotics (Chusri *et al*., 2009), mainly through efflux pump inhibition. Although still poorly explored, inhibition of regulatory mechanisms that control bacterial virulence functions represents a promising strategy for antibiotic adjuvant therapy. The non-essential character of these functions may significantly reduce the development of resistance. The continuous advances in the development of new and potent high-throughput technologies will definitively allow the discovery of new compounds with antibiotic adjuvant activity.

## Conflict of interest

None declared.

## References

[b1] Alekshun MN, Levy SB (2007). Molecular mechanisms of antibacterial multidrug resistance. Cell.

[b2] Aoki N, Tateda K, Kikuchi Y, Kimura S, Miyazaki C, Ishii Y (2009). Efficacy of colistin combination therapy in a mouse model of pneumonia caused by multidrug-resistant *Pseudomonas aeruginosa*. J Antimicrob Chemother.

[b3] Balfour JA, Bryson HM, Brogden RN (1996). Imipenem/cilastatin: an update of its antibacterial activity, pharmacokinetics and therapeutic efficacy in the treatment of serious infections. Drugs.

[b4] Barnes AI, Herrero IL, Albesa I (2005). New aspect of the synergistic antibacterial action of ampicillin and gentamicin. Int J Antimicrob Agents.

[b5] Barraud N, Hassett DJ, Hwang SH, Rice SA, Kjelleberg S, Webb JS (2006). Involvement of nitric oxide in biofilm dispersal of *Pseudomonas aeruginosa*. J Bacteriol.

[b6] Bernal P, Llamas MA (2012). Promising biotechnological applications of antibiofilm exopolysaccharides. Microb Biotechnol.

[b7] Bhardwaj AK, Mohanty P (2012). Bacterial efflux pumps involved in multidrug resistance and their inhibitors: rejuvinating the antimicrobial chemotherapy. Recent Pat Antiinfect Drug Discov.

[b8] Brown AG, Butterworth D, Cole M, Hanscomb G, Hood JD, Reading C, Rolinson GN (1976). Naturally-occurring beta-lactamase inhibitors with antibacterial activity. J Antibiot (Tokyo).

[b9] Chanbusarakum P, Murray PR (1978). Analysis of the interactions between piperacillin, ticarcillin, or carbenicillin and aminoglycoside antibiotics. Antimicrob Agents Chemother.

[b10] Chusri S, Villanueva I, Voravuthikunchai SP, Davies J (2009). Enhancing antibiotic activity: a strategy to control *Acinetobacter* infections. J Antimicrob Chemother.

[b12] D'Costa VM, McGrann KM, Hughes DW, Wright GD (2006). Sampling the antibiotic resistome. Science.

[b11] D'Costa VM, King CE, Kalan L, Morar M, Sung WW, Schwarz C (2011). Antibiotic resistance is ancient. Nature.

[b13] Drawz SM, Bonomo RA (2010). Three decades of beta-lactamase inhibitors. Clin Microbiol Rev.

[b14] Ejim L, Farha MA, Falconer SB, Wildenhain J, Coombes BK, Tyers M (2011). Combinations of antibiotics and nonantibiotic drugs enhance antimicrobial efficacy. Nat Chem Biol.

[b15] Feder M, Purta E, Koscinski L, Cubrilo S, Maravic Vlahovicek G, Bujnicki JM (2008). Virtual screening and experimental verification to identify potential inhibitors of the ErmC methyltransferase responsible for bacterial resistance against macrolide antibiotics. Chem Med Chem.

[b16] Gordon NC, Png K, Wareham DW (2010). Potent synergy and sustained bactericidal activity of a vancomycin-colistin combination versus multidrug-resistant strains of *Acinetobacter baumannii*. Antimicrob Agents Chemother.

[b17] Hemaiswarya S, Doble M (2009). Synergistic interaction of eugenol with antibiotics against Gram negative bacteria. Phytomedicine.

[b18] Hemaiswarya S, Doble M (2010). Synergistic interaction of phenylpropanoids with antibiotics against bacteria. J Med Microbiol.

[b19] Hugonnet JE, Tremblay LW, Boshoff HI, Barry CE, Blanchard JS (2009). Meropenem-clavulanate is effective against extensively drug-resistant *Mycobacterium tuberculosis*. Science.

[b20] Kalan L, Wright GD (2011). Antibiotic adjuvants: multicomponent anti-infective strategies. Expert Rev Mol Med.

[b21] Kastoris AC, Rafailidis PI, Vouloumanou EK, Gkegkes ID, Falagas ME (2010). Synergy of fosfomycin with other antibiotics for Gram-positive and Gram-negative bacteria. Eur J Clin Pharmacol.

[b22] Kolodkin-Gal I, Romero D, Cao S, Clardy J, Kolter R, Losick R (2010). d-amino acids trigger biofilm disassembly. Science.

[b23] Lee S, Hinz A, Bauerle E, Angermeyer A, Juhaszova K, Kaneko Y (2009). Targeting a bacterial stress response to enhance antibiotic action. Proc Natl Acad Sci USA.

[b24] Leflon-Guibout V, Speldooren V, Heym B, Nicolas-Chanoine M (2000). Epidemiological survey of amoxicillin-clavulanate resistance and corresponding molecular mechanisms in *Escherichia coli* isolates in France: new genetic features of bla(TEM) genes. Antimicrob Agents Chemother.

[b25] Moellering RC, Eliopoulos GM, Allan JD (1986). Beta-lactam/aminoglycoside combinations: interactions and their mechanisms. Am J Med.

[b26] Molina-Quiroz RC, Munoz-Villagran CM, Torre E, Tantalean JC, Vasquez CC, Perez-Donoso JM (2012). Enhancing the antibiotic antibacterial effect by sub lethal tellurite concentrations: tellurite and cefotaxime act synergistically in *Escherichia coli*. PLoS ONE.

[b27] Nguyen HT, Wolff KA, Cartabuke RH, Ogwang S, Nguyen L (2010). A lipoprotein modulates activity of the MtrAB two-component system to provide intrinsic multidrug resistance, cytokinetic control and cell wall homeostasis in *Mycobacterium*. Mol Microbiol.

[b28] Pagès JM, Amaral L (2009). Mechanisms of drug efflux and strategies to combat them: challenging the efflux pump of Gram-negative bacteria. Biochim Biophys Acta.

[b29] Poole K (2012). Stress responses as determinants of antimicrobial resistance in Gram-negative bacteria. Trends Microbiol.

[b30] Reeves DS (1971). Sulphamethoxazole-trimethoprim: the first two years. J Clin Pathol.

[b31] Rendueles O, Kaplan JB, Ghigo JM Antibiofilm polysaccharides. Environ Microbiol.

[b32] Stewart PS, Costerton JW (2001). Antibiotic resistance of bacteria in biofilms. Lancet.

[b33] Taylor PL, Rossi L, De Pascale G, Wright GD (2012). A forward chemical screen identifies antibiotic adjuvants in *Escherichia coli*. ACS Chem Biol.

[b34] Van Bambeke F, Pagès JM, Lee VJ, Atta-ur-Rahman, Choudhary MI (2010). Inhibitors of bacterial efflux pumps as adjuvants in antibacterial therapy and diagnostic tools for detection of resistance by efflux.

[b35] Vong K, Tam IS, Yan X, Auclair K (2012). Inhibitors of aminoglycoside resistance activated in cells. ACS Chem Biol.

